# The Triglyceride-Glucose Index and Obesity-Related Risk of End-Stage Kidney Disease in Austrian Adults

**DOI:** 10.1001/jamanetworkopen.2021.2612

**Published:** 2021-03-31

**Authors:** Josef Fritz, Wolfgang Brozek, Hans Concin, Gabriele Nagel, Julia Kerschbaum, Karl Lhotta, Hanno Ulmer, Emanuel Zitt

**Affiliations:** 1 Department of Medical Statistics, Informatics and Health Economics, Innsbruck Medical University, Innsbruck, Austria; 2 Agency for Preventive and Social Medicine, Bregenz, Austria; 3 Institute of Epidemiology and Medical Biometry, Ulm University, Ulm, Germany; 4 Department of Internal Medicine IV (Nephrology and Hypertension), Innsbruck Medical University, Innsbruck, Austria; 5 Department of Internal Medicine III (Nephrology and Dialysis), Academic Teaching Hospital Feldkirch, Feldkirch, Austria; 6 Vorarlberg Institute for Vascular Investigation and Treatment, Academic Teaching Hospital Feldkirch, Feldkirch, Austria

## Abstract

**Importance:**

It is unknown whether the triglyceride-glucose (TyG) index as a measure of insulin resistance is associated with the risk of developing end-stage kidney disease (ESKD). Because individuals who are overweight or obese often develop insulin resistance, mediation of the association between body mass index (BMI) and ESKD risk through the TyG index seems plausible but has not been investigated.

**Objective:**

To evaluate whether the TyG index is associated with ESKD risk and, if so, to what extent the TyG index mediates the association between BMI and ESKD.

**Design, Setting, and Participants:**

A total of 176 420 individuals were recruited during routine health examinations to participate in the Austrian Vorarlberg Health Monitoring and Promotion Program (VHM&PP), a prospective, population-based cohort study with participant enrollment between January 1, 1988, and June 30, 2005, and a mean follow-up of 22.7 years. Data analysis was conducted from March 1, 2020, to September 30, 2020.

**Exposures:**

Body mass index and the logarithmized product of fasting triglyceride and glucose concentrations (TyG index), as determined during the baseline health examination.

**Main Outcomes and Measures:**

End-stage kidney disease, as indicated by initiation of kidney replacement therapy, either dialysis or kidney transplantation.

**Results:**

Of the 176 420 participants, 94 885 were women (53.8%); mean (SD) age was 42.5 (15.4) years. During a mean (SD) follow-up of 22.7 (6.9) years, 454 (0.3%) participants developed ESKD and 35 234 (20.0%) died. In multivariable-adjusted Cox proportional hazards models, the TyG index was significantly associated with the risk of ESKD, both with (hazard ratio [HR] per 1-SD increase, 1.68; 95% CI, 1.56-1.82) and without (HR per 1-SD increase, 1.79; 95% CI, 1.66-1.93) the inclusion of BMI as a covariate. Mediation analysis using a newly proposed 2-stage regression method for survival data showed that a 5-point increase in BMI increased the risk of ESKD by 58% (HR [total association], 1.58; 95% CI, 1.43-1.75), and that 41.7% of the total association (95% CI, 31.6%-51.8%) was mediated through the TyG index (HR [indirect association], 1.21; 95% CI, 1.18-1.25).

**Conclusions and Relevance:**

This study found that the TyG index appeared to be associated with ESKD risk and mediates nearly half of the total association between BMI and ESKD in the general population. Public health efforts aiming at the reduction of body weight might decrease the kidney sequelae of insulin resistance and the burden of ESKD.

## Key Points

**Question:**

To what extent does the triglyceride-glucose index, a novel measure of insulin resistance, explain the association between body mass index and end-stage kidney disease risk?

**Findings:**

In this population-based cohort study of 176 420 Austrian participants, the triglyceride-glucose index was significantly associated with incident end-stage kidney disease risk. Approximately 40% of the association between body mass index and end-stage kidney disease was mediated through the triglyceride-glucose index.

**Meaning:**

The findings of this study appear to support the hypothesis of insulin resistance being an important intermediate in the association between obesity and end-stage kidney disease.

## Teaser

This cohort study examines the use of the triglyceride-glucose index as a measure of insulin resistance in individuals who are overweight or obese.

## Introduction

Chronic kidney disease (CKD) affects approximately 10% to 15% of the adult general population worldwide. The numbers are increasing owing to the growing aging population and lifestyle changes associated with an increased prevalence of obesity, hypertension, and diabetes.^1,2,3^ The increase in obesity prevalence is reported worldwide and is estimated to further increase by 40% by 2027.^4^ Globally, obesity is associated with a 36% increased risk of CKD in the general population.^5,6^ Individuals who are obese have a more than 3-fold higher risk of developing end-stage kidney disease (ESKD) than those with normal body weight.^7,8,9^ End-stage kidney disease and the subsequent kidney replacement therapy represent a major burden for individuals and health care systems.^10,11^

Although multiple neurohumoral, metabolic, and hemodynamic components have been suggested as factors in an association between obesity and kidney disease, the exact mechanisms are still not fully understood.^12,13^ Decreased insulin sensitivity might be one component, favoring a hyperglycemic state that eventually results in diabetes and diabetic kidney disease. In addition to and independent of the later development of diabetes, insulin resistance per se is associated with glomerular hyperfiltration, sodium retention, defective tubular reabsorption, tissue inflammation, and fibrosis.^14,15,16^

The logarithmized product of fasting triglyceride and glucose levels (triglyceride-glucose [TyG] index) has been shown to be a simple measure of insulin resistance.^17^ The TyG index correlates with the euglycemic-hyperinsulinemic clamp test, and its validity is similar to the homeostatic model assessment insulin resistance index.^18^ Owing to its easy availability and good performance, the TyG index can be conveniently used in large-scale epidemiologic studies as a simple surrogate measure for insulin resistance.

To our knowledge, no studies have been conducted on the association between the TyG index and ESKD risk and the role of the TyG index in the association between BMI and ESKD risk. Assuming that decreased insulin sensitivity plays a substantial role in the association between obesity and kidney disease, we hypothesized that the TyG index is associated with risk of ESKD and that part of the association between BMI and ESKD is mediated through the TyG index. Using data from the Vorarlberg Health Monitoring and Promotion Program (VHM&PP), a low-risk population-based cohort followed up for as long as 30 years, we quantified these associations and calculated the proportion mediated through the TyG index.

## Methods

### Data Source and Study Population

The VHM&PP is a large, ongoing, population-based risk factor surveillance program in Vorarlberg, the westernmost province of Austria. Every adult residing in Vorarlberg was invited to participate, and a screening examination was performed by local general practitioners according to a standard protocol. Between January 1985 and June 2005, 99 894 female and 85 473 male residents older than 18 years (approximately two-thirds of the adult population of Vorarlberg) were enrolled in the VHM&PP. During the screening examination, height and weight (in light clothing) were measured by medical staff, smoking status was determined, and a blood sample was obtained. A more detailed description of the program is reported elsewhere.^9,19,20^

Because an overnight fast was part of the protocol only from January 1, 1988, onward, we excluded 8073 participants (4.4%) who did not have an examination with a blood sample obtained in fasting status. Of the remaining participants, we excluded another 874 (0.5%) owing to missing BMI, glucose, or triglyceride values, resulting in a final analysis population of 176 420 participants initially free of ESKD at the baseline examination, with complete information on exposure, mediator, outcome variables, and covariates.

Outcome data were obtained by linking the VHM&PP database with the Austrian Dialysis and Transplant Registry and the National Mortality Registry. The Austrian Dialysis and Transplant Registry collects data, which are provided by the Austrian dialysis and transplant centers, on all patients receiving chronic kidney replacement therapy (hemodialysis, peritoneal dialysis, and kidney transplantation) in Austria since 1964 with an almost complete follow-up.^21^ Data analysis was conducted from March 1, 2020, to September 30, 2020.

All study procedures were performed in accordance with the Declaration of Helsinki^22^ and relevant guidelines. Institutional review board approval for the study was obtained from the ethics committee of the State of Vorarlberg. Written informed consent was obtained from all VHM&PP participants, and all patients registered in the Austrian Dialysis and Transplant Registry signed a declaration of consent to permit their data to be transferred to the registry. This study followed the Strengthening the Reporting of Observational Studies in Epidemiology (STROBE) reporting guideline for cohort studies.^23^

### Definitions of Exposure, Mediator, and Outcome

Body mass index was calculated from height and weight records as weight in kilograms divided by height in meters squared and categorized as underweight (BMI<18.5), normal weight (BMI 18.5-<25), overweight (BMI 25-<30), and obesity (BMI≥30) according to the World Health Organization definition.^24^ The TyG index was calculated as ln [fasting triglycerides (milligrams per deciliter) × fasting blood glucose (milligrams per deciliter) / 2)^17^ and split into quartiles. The outcome ESKD was defined as initiation of kidney replacement therapy, either dialysis or kidney transplantation. Follow-up began after the baseline health examination and ended at the diagnosis of ESKD or at the occurrence of the censoring events death or end of the observation period (December 31, 2018), whichever occurred first.

### Statistical Analysis

Only exposure, mediator, and covariate data from each individual´s first health examination were included in the analysis. We tabulated participant characteristics both overall and stratified by TyG index quartiles, using Cochrane-Armitage tests and linear regression *t* tests using quartile numbers (1, 2, 3, and 4) to test for trends over TyG index quartiles. The association between the TyG index (both as a linear term and as quartiles) and risk of ESKD was modeled using Cox proportional hazards regressions. Linear trends over TyG index quartiles was assessed using Wald tests of a linear association of the quartiles as a numeral (1-4) with the risk of ESKD. For assessing mediation of the association between BMI and ESKD through the TyG index, we applied the 2-stage regression method for survival data proposed by VanderWeele.^25^ In brief, 2 regression models are fit to the data, 1 modeling the mediator and the other modeling the outcome; parameter estimates and SEs of these 2 separate models are combined according to the formulas given therein^25^ to obtain estimates and SEs for effect size of mediation. We modeled the outcome (ESKD) using Cox proportional hazards regression models with time since baseline examination as the underlying time variable, and the mediator (TyG index) using linear regressions. We used the TyG index as a linear term because a Cox proportional hazards regression model using the TyG index categorized into quartiles confirmed a linear association between the TyG index and risk of ESKD. All models were adjusted for age, sex, and smoking status because these variables are established factors in the outcome ESKD,^8^ without inclusion of interaction terms. We conducted analogous mediation analyses using BMI categories instead of continuous BMI, restricting analysis to men and women, truncating the follow-up at 10 years, and using triglyceride and glucose levels as mediators instead of the TyG index.

Assuming associations between variables as shown in the directed acyclic graph in the Figure,^26^ and assuming that age, sex, and smoking status account for the majority of confounding, VanderWeele’s method decomposes the total effect of BMI on ESKD (expressed as the hazard ratio [HR] per 5-point increase in BMI, or as the HR vs the reference normal weight for BMI categories) into 2 components: the natural indirect effect size (ie, the effect size of BMI that is due to mediation through the TyG index), and the natural direct effect size (ie, the effect size of BMI not explained through the mediator).^27,28^ Because these estimates are based on observational data, we term the estimates the total, indirect, and direct associations. The proportion of the association between BMI and ESKD mediated through the TyG index as a measure of the contribution of the natural indirect association with the total association was calculated on the log-transformed HR scale as log(indirect association HR)/log(total association HR), since HRs are additive on this scale. Both the 95% CIs for estimates of the total, natural indirect, and natural direct associations and the proportion mediated were calculated based on SEs derived from the delta method.^29^ Additional information on our model is given in the eMethods in the Supplement.

**Figure.**
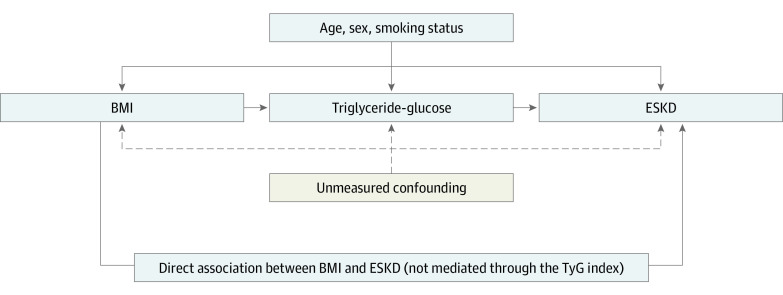
Directed Acyclic Graph Depicting the Hypothesized Associations Between Exposure Body Mass Index (BMI), Mediator Triglyceride-Glucose Index (TyG), Outcome End-Stage Kidney Disease (ESKD), and the Relevant Confounders Age, Sex, and Smoking Status Possible other mechanisms through which the association between BMI and ESKD is mediated, such as hypertension, hypercholesterolemia, and/or hyperuricemia, are contained in the direct association between BMI and ESKD. All statistical models were based on this structure and were adjusted for age, sex, and smoking status. Because blood pressure, cholesterol, and uric acid levels represent alternative pathways potentially mediating parts of the association between BMI and ESKD, these variables were not entered as covariates in our models according to the theory of causal graphs.^26^ The possibility of unmeasured confounding, which can never be ruled out in observational research, is indicated with dashed arrows.

All statistical tests were 2-sided at a significance level of *P* < .05. Mediation analysis was conducted in SAS, version 9.4 (SAS Institute Inc), using the macro *%mediation* by Valeri and VanderWeele,^30^ and the rest of the analyses were conducted in R, version 3.5.1 (R Foundation).

## Results

The analyzed cohort included 176 420 participants (94 885 women [53.8%], 81 535 men [46.2%], mean [SD] baseline age, 42.5 [15.4] years) initially free of ESKD, of whom 454 (0.3%) developed ESKD and 35 234 (20.0%) died over a mean follow-up of 22.7 (6.9) years (ie, 4 001 979 person-years of follow-up) (Table 1). Mean age at the start of kidney replacement therapy was 65.2 (12.7) years, and mean time from baseline until ESKD was 13.8 (7.6) years. Mean BMI was 24.9 (4.3); 56 136 (31.8%) of the participants were overweight, and 20 275 (11.5%) were obese. Mean fasting glucose level was 88.3 (23.4) mg/dL (to convert to millimoles per liter, multiply by 0.0555) and triglyceride level was 132.7 (97.4) mg/dL (to convert to millimoles per liter, multiply by 0.0113) yielding a mean (SD) TyG index of 8.5 (0.6) (Table 1).

**Table 1.** Participant Characteristics of the VHM&PP Cohort, Overall and Stratified by Quartiles of the TyG Index^a^

Characteristic^b^,^c^	No. (%)				
	Total	TyG index^d^			
		Quartile 1 (<8.02)	Quartile 2 (8.02-8.38)	Quartile 3 (8.39-8.81)	Quartile 4 (≥8.82)
No.	176 420	44 194	44 033	44 070	44 123
Baseline age, mean (SD), y	42.5 (15.4)	36.9 (13.2)	41.0 (15.2)	44.1 (15.9)	48.1 (15.1)
Sex					
Male	81 535 (46.2)	14 944 (33.8)	17 939 (40.7)	21 266 (48.3)	27 386 (62.1)
Female	94 885 (53.8)	29 250 (66.2)	26 094 (59.3)	22 804 (51.7)	16 737 (37.9)
Smoking status					
Nonsmoker	123 532 (70.0)	33 583 (76.0)	31 722 (72.0)	30 327 (68.8)	27 900 (63.2)
Ex-smoker	12 520 (7.1)	2421 (5.5)	2717 (6.2)	3126 (7.1)	4256 (9.6)
Smoker	40 368 (22.9)	8190 (18.5)	9594 (21.8)	10 617 (24.1)	11 967 (27.1)
BMI, mean (SD)	24.9 (4.3)	22.8 (3.4)	24.1 (3.9)	25.3 (4.2)	27.3 (4.4)
Weight					
Underweight (BMI, <18.5)	5411 (3.1)	2528 (5.7)	1676 (3.8)	907 (2.1)	300 (0.7)
Normal (BMI, 18.5-24.9)	94 598 (53.6)	32 230 (72.9)	27 121 (61.6)	21 869 (49.6)	13 378 (30.3)
Overweight (BMI, 25.0-29.9)	56 136 (31.8)	7970 (18.0)	12 059 (27.4)	15 821 (35.9)	20 286 (46.0)
Obese (BMI, ≥30.0)	20 275 (11.5)	1466 (3.3)	3177 (7.2)	5473 (12.4)	10 159 (23.0)
Fasting glucose, mean (SD), mg/dL	88.3 (23.4)	77.5 (12.6)	84.7 (12.6)	88.3 (14.4)	102.7 (36.0)
Diabetes^e^	6511 (3.7)	39 (0.1)	228 (0.5)	833 (1.9)	5411 (12.3)
Fasting triglycerides, mg/dL	132.7 (97.4)	61.9 (17.7)	88.5 (17.7)	123.9 (26.5)	247.8 (141.6)
Hypertriglyceridemia^f^	22 850 (13.0)	0	10 (0.0)	420 (1.0)	22 420 (50.8)
TyG index, mean (SD)^d^	8.5 (0.6)	7.7 (0.2)	8.2 (0.1)	8.6 (0.1)	9.3 (0.4)
Blood pressure, mean (SD), mm Hg					
Systolic	129.7 (20.6)	122.2 (17.3)	127.1 (19.3)	131.4 (20.6)	138.1 (21.5)
Diastolic	80.5 (11.1)	77.0 (9.9)	79.3 (10.5)	81.3 (11.0)	84.4 (11.4)
Hypertension^g^	65 617 (37.2)	9488 (21.5)	13 836 (31.4)	17 989 (40.8)	24 304 (55.1)
Fasting total cholesterol, mean (SD), mg/dL	212.4 (46.3)	189.2 (34.7)	204.6 (38.6)	220.1 (42.5)	239.4 (50.2)
Hypercholesterolemia^h^	45 458 (25.8)	3808 (8.6)	8150 (18.5)	12 889 (29.2)	20 611 (46.7)
Uric acid, mean (SD), md/dL	5.3 (1.4)	4.8 (1.3)	5.1 (1.3)	5.3 (1.3)	5.8 (1.5)
Hyeruricemia^i^	17 565 (14.4)	1 382 (6.0)	2 515 (9.0)	4 285 (13.3)	9 383 (24.6)
γ-glutamyltransferase, mean (SD), U/L	56.4 (84.7)	36.9 (41.2)	45.5 (56.2)	55.6 (74.5)	87.6 (129.6)
Elevated γ-glutamyltransferase^j^	60 058 (34.1)	7 999 (18.1)	11 813 (26.8)	15 909 (36.1)	24 337 (55.2)
Follow-up, mean (SD), y	22.7 (6.9)	23.6 (6.1)	23.1 (6.6)	22.6 (7.0)	21.4 (7.5)
Death by any cause during follow-up	35 234 (20.0)	4299 (9.7)	7356 (16.7)	9880 (22.4)	13 699 (31.0)
ESKD during follow-up	454 (0.3)	38 (0.1)	67 (0.2)	99 (0.2)	250 (0.6)

Abbreviations: BMI (calculated as weight in kilograms divided by height in meters squared); ESKD, end-stage kidney disease; TyG, triglyceride-glucose index; VHM&PP, Vorarlberg Health Monitoring and Promotion Program.

SI conversion factors: To convert cholesterol to millimoles per liter, multiply by 0.0259; γ-glutamyltransferase to microkatals per liter, 0.0167; glucose to millimoles per liter, 0.0555; triglycerides to millimoles per liter, 0.0113; and uric acid to millimoles per liter, 0.0595.

^a^ Trend tests across TyG index quartiles (Cochrane-Armitage tests for categorical variables, linear regression *t* tests using quartile numbers (1, 2, 3, 4) for continuous variables) yielded *P* < .001 for all participant characteristics variables.

^d^ TyG index was calculated as ln (triglycerides [milligrams per deciliter] × blood glucose [milligrams per deciliter] / 2).

^e^ Diabetes was defined as fasting blood glucose levels greater than 6.9 mmol/L.

^f^ Hypertriglyceridemia was defined as fasting triglycerides greater than or equal to 2.3 mmol/L.

^g^ Hypertension was defined as systolic blood pressure greater than or equal to 140 mm Hg or diastolic blood pressure greater than or equal to 90 mm Hg.

^h^ Hypercholesterolemia was defined as fasting total cholesterol level greater than or equal to 6.2 mmol/L.

^i^ Hyperuricemia was defined as uric acid level greater than 340 μmol/L in women and greater than 420 μmol/L in men.

^j^ Elevated γ-glutamyltransferase was defined as levels greater than or equal to 36 IU/L in women and greater than or equal to 61 IU/L in men.

Stratification of participant characteristics by TyG index quartiles showed that the TyG index was associated with higher BMI and increased ESKD incidence, but also with older age, male sex, smoking, higher blood pressure, and cholesterol, γ-glutamyltransferase, and uric acid levels (all *P* < .001 for trend) (Table 1). The association of BMI with the TyG index remained significant after adjusting for sex, age, and smoking status (adjusted β per 5-point increase in BMI, 0.229; 95% CI, 0.226-0.232; adjusted *R*^2^ = 0.22). In Cox proportional hazards models adjusted for sex, age, and smoking status, the TyG index was significantly associated with the risk of ESKD, both with (HR per 1-SD increase, 1.68; 95% CI, 1.56-1.82) and without (HR per 1-SD increase 1.79; 95% CI, 1.66-1.93) the inclusion of BMI as a covariate (Table 2). Analyses using the TyG index categorized into quartiles showed a linear association between the TyG index and the risk of ESKD (Table 2).

**Table 2.** Risk of ESKD by TyG Index

Variable	TyG index	HR (95% CI)	*P* value
Adjusted			
Age, sex, and smoking status	Continuous^a^	1.79 (1.66-1.93)	<.001
	Quartiles		
	Q1 (<8.02)	1 [Reference]	<.001^b^
	Q2 (8.02-8.38)	1.43 (0.96-2.13)	
	Q3 (8.39-8.81)	1.81 (1.24-2.64)	
	Q4 (≥8.82)	3.92 (2.76-5.57)	
Age, sex, and smoking status, plus BMI	Continuous^a^	1.68 (1.56-1.82)	<.001
	Q1 (<8.02)	1 [Reference]	<0001^b^
	Q2 (8.02-8.38)	1.32 (0.88-1.96)	
	Q3 (8.39-8.81)	1.55 (1.06-2.27)	
	Q4 (≥8.82)	3.02 (2.10-4.33)	

Abbreviations: BMI, body mass index (calculated as weight in kilograms divided by height in meters squared); ESKD, end-stage kidney disease; HR, hazard ratio; Q, quartile; TyG, triglyceride-glucose index.

^a^ HR given per SD increase.

^b^ *P* values for trend, obtained from Wald tests of a linear association of the quartile as a numeral (1-4) with risk of ESKD.

Mediation analysis showed that an increase in BMI by 5 points increased the risk of ESKD by 58% (HR [total association], 1.58; 95% CI, 1.43-1.75), and that nearly half of the total association (41.7%; 95% CI, 31.6%-51.8%) was mediated through the TyG index (HR [indirect association], 1.21; 95% CI, 1.18-1.25) (Table 3). Exclusion of underweight individuals from the analysis left the results virtually unchanged. Analysis by BMI categories yielded a total association HR of 1.48 (95% CI, 1.19-1.85) for overweight vs the reference normal weight, which increased to 2.73 (95% CI, 2.12-3.53) for the obesity group. Proportions mediated were 57.5% (95% CI, 23.7%-91.4%) for overweight, and 49.8% (95% CI, 35.5%-64.2%) for obesity.

**Table 3.** Decomposition of the Total Association Between Body Mass Index and the Risk of ESKD Into Direct and Indirect Associations Mediated by the TyG Index

Variable	ESKD incident cases/persons, No.	Association^a^						Proportion mediated, % (95% CI)
		Total^b^		Indirect		Direct		
		HR (95% CI)	*P* value	HR (95% CI)	*P* value	HR (95% CI)	*P* value	
Model/group								
BMI continuous^a^	454/176 420	1.58 (1.43-1.75)	<.001	1.21 (1.18-1.25)	<.001	1.31 (1.18-1.45)	<.001	41.7 (31.6-51.8)
BMI continuous (≥18.5)^a^	451/171 009	1.59 (1.44-1.76)	<.001	1.22 (1.18-1.25)	<.001	1.31 (1.18-1.45)	<.001	42.3 (32.1-52.6)
Normal weight	141/94 598	1 [Reference]						
Overweight	196/56 136	1.48 (1.19-1.85)	.001	1.25 (1.20-1.31)	<.001	1.18 (0.94-1.49)	.15	57.5 (23.7-91.4)
Obesity	114/20 275	2.73 (2.12-3.53)	<.001	1.65 (1.51-1.80)	<.001	1.66 (1.26-2.18)	<.001	49.8 (35.5-64.2)
BMI continuous^a^								
Men	286/81 535	1.68 (1.45-1.93)	<.001	1.23 (1.17-1.29)	<.001	1.37 (1.18-1.59)	<.001	39.5 (26.6-52.4)
Women	168/94 885	1.54 (1.35-1.77)	<.001	1.25 (1.20-1.29)	<.001	1.24 (1.08-1.43)	.003	50.7 (33.7-67.8)
Participants with baseline fasting glucose ≤126 mg/dL only								
BMI continuous^a^	365/169 909	1.47 (1.31-1.65)	<.001	1.12 (1.08-1.16)	<.001	1.31 (1.16-1.48)	<.001	29.6 (18.1-41.1)
Normal weight	125/92 807	1 [Reference]						
Overweight	161/53 494	1.42 (1.12-1.80)	.004	1.16 (1.10-1.22)	<.001	1.22 (0.96-1.57)	.11	42.3 (11.1-73.5)
Obesity	76/18 263	2.32 (1.73-3.11)	<.001	1.37 (1.23-1.53)	<.001	1.69 (1.23-2.32)	.001	37.6 (21.3-53.9)
Follow-up truncated at 10 y								
BMI continuous^a^	157/176 420	1.26 (1.05-1.52)	.01	1.25 (1.19-1.31)	<.001	1.01 (0.83-1.23)	.92	95.6 (15.3-176.0)

Abbreviations: BMI, body mass index (calculated as weight in kilograms divided by height in meters squared); DAG, directed acyclic graph; ESKD, end-stage kidney disease; HR, hazard ratio; TyG, triglyceride-glucose index.

SI conversion factor: To convert glucose to millimoles per liter, multiply by 0.0555.

^a^ Hazard ratios given per 5-point increase.

^b^ Decomposition of total associations into natural indirect and natural direct associations was done according to the 2-stage regression method proposed by VanderWeele^25^ and performed with the SAS macro provided by Valeri and VanderWeele.^30^ Confidence intervals were calculated according to the delta method procedure. All models were adjusted for age, sex, and smoking status as depicted in the DAG in the Figure.

Subgroup analyses revealed similar results across men and women. Excluding participants with baseline fasting plasma glucose levels greater than 125 g/dL slightly attenuated the total association (HR, 1.47; 95% CI, 1.31-1.65) and the indirect association (HR, 1.12; 95% CI, 1.08-1.16) between BMI and ESKD risk, with 29.6% (95% CI, 18.1%-41.1%) mediated by the TyG index (Table 3). Truncation of the follow-up at 10 years resulted in a marked reduction in ESKD event numbers (n = 157). The total association between BMI and ESKD risk was attenuated compared with our main model using the complete follow-up (HR, 1.26; 95% CI, 1.05-1.52); conversely, the proportion mediated increased (95.6%; 95% CI, 15.3%-176.0%) (Table 3).

## Discussion

In this large, observational, population-based cohort study, we found that the TyG index was independently associated with an increased risk of ESKD and nearly half (41.7%) of the total association between BMI and the risk of ESKD was mediated through the TyG index.

Various population-based studies have described an association between higher BMI and the development of CKD^31,32,33,34^ as well as a more rapid decline of kidney function.^35^ Consistently, epidemiologic studies have indicated that a higher BMI is an independent estimator for future ESKD.^7,8,9,36,37,38^ Our study supports these findings and noted that an increase in BMI by 5 points increased the risk of ESKD by 58%. In terms of BMI categories, the risk for ESKD increased by 48% in participants who were overweight and almost tripled (HR, 2.73) in participants with obesity compared with the reference normal weight populations.

Individuals who are overweight or obese are more likely to develop insulin resistance indicating early impaired glucose metabolism.^39^ Cross-sectional studies have shown an association of insulin resistance with CKD independent of diabetes, which was even greater in the presence of obesity.^40,41^ Therefore, insulin resistance might be a potential important mediator of the association between BMI and the risk of ESKD.

To our knowledge, our study is the first to examine the association between the TyG index as a validated measure of insulin resistance and ESKD risk and analyze its mediating role in BMI-related ESKD risk. In our study, using multivariable-adjusted models, the baseline TyG index was independently associated with an increased risk of ESKD over a mean follow-up of 22.7 years, and 41.7% of the total association between BMI and ESKD risk was mediated through the TyG index. When truncating the follow-up at 10 years, the mediating association with the TyG index was even greater; however, the total association between BMI and ESKD was attenuated. A reason for this observation might be the marked difference regarding baseline age in cases of ESKD occurring within 10 years after baseline (mean age, 55.3 years) vs cases occurring beyond 10 years (mean age, 49.4 years). Another possible explanation for this instance of reverse epidemiologic findings (ie, a markedly different pattern of BMI for short-term ESKD incidence than for long-term incidence) is that participants developing ESKD in the short or medium run are already likely to present with deteriorating health, with associated weight loss and lower BMI. The observation of a possible reverse epidemiology in the short term also highlights the importance of a sufficiently long follow-up when studying the long-term consequences of high BMI on ESKD risk.

Our study results provide epidemiologic support for the biologically plausible hypothesis that insulin resistance plays an important role in the pathway between obesity and ESKD. It is conceivable that the association between the TyG index and ESKD risk and the association of BMI with ESKD mediated through the TyG index at baseline can in part be explained by the development of diabetes and diabetic kidney disease during the long follow-up time. Investigating the role of the 2 single components of the TyG index—fasting glucose and triglyceride levels—separately as mediators of the association between BMI and ESKD revealed that both triglycerides and glucose were only weak mediators (proportion mediated through triglycerides: 10.8%; 95% CI, 7.8%-13.8%, and through glucose: 11.6%; 95% CI, 8.6%-14.6%) (eTable in the Supplement), whereas the TyG index as an entity mediated 42% of the total association between BMI and ESKD, indicating that the whole (TyG index) is more than the sum of its parts (triglycerides and glucose). This observation also supports our hypothesis that the TyG index is biologically meaningful and a valid marker of insulin resistance.

Our findings have clinical and public health implications, because the epidemic of obesity is accompanied by a growing number of patients with CKD worldwide.^5,42,43^ Obesity clearly is a modifiable risk factor and a considerable proportion of ESKD and diabetes may be prevented if the general population maintained a normal BMI. To our knowledge, no lifestyle intervention studies focusing on weight reduction in persons who are overweight or obese have been carried out with ESKD as an end point. However, bariatric surgery as a weight-reducing intervention was found to result in a significant reduction of insulin resistance.^44,45^ The long-term incidence of ESKD and stage 4 CKD can be significantly reduced by successful bariatric surgery in patients with obesity, as has been reported recently in a post hoc analysis of the Swedish Obese Subjects study.^46^ These studies indirectly support the idea of a causal pathway from obesity to insulin resistance and CKD with ESKD, emphasizing the importance of weight reduction to maintain kidney health.

### Strengths and Limitations

Our findings are noteworthy because they are based on a large representative cohort of a central European general population with a long follow-up, which is necessary to meaningfully study longitudinal associations, such as obesity-related ones, on the kidney. In addition, carefully selected and standardized measures of study exposure and outcome variables allowed precise estimation of the measures of association and mediation. Furthermore, we applied a new analytical tool developed in the counterfactual framework that allows, in contrast to traditional methods for mediation analysis, a mathematically consistent decomposition of the total association into direct and indirect associations with clear interpretations.^25,28^

The study has limitations. First, we used BMI to determine overweight and obesity. Although widely used and easy to calculate, BMI is a poor estimate of proportion and distribution of fat mass. We lacked alternative parameters, such as waist circumference,^47^ waist-to-hip ratio,^48^ or body fat composition analysis,^49^ which might more accurately determine visceral fat and represent even more sensitive estimators of kidney sequelae. Second, in the VHM&PP cohort, baseline data on kidney function were not available. However, given the long period of 13.8 years between baseline examination and development of ESKD, we believe that it is unlikely that we have included a significant number of patients with relevant kidney disease at the time of BMI measurement and TyG index calculation. Third, our data and results refer to a low-risk, general population-based cohort of White individuals, limiting generalization including different age groups and ethnicities. Fourth, although the possibility of unmeasured confounding cannot be ruled out, the magnitude of the observed effect sizes makes it unlikely that unmeasured confounding could completely explain our observed associations.

## Conclusions

Our findings suggest that the TyG index can be used to identify individuals at risk of developing ESKD and that the TyG index mediates nearly half of the total association between BMI and ESKD in our general population cohort. Public health efforts aiming at the reduction of body weight might decrease the kidney sequelae of insulin resistance and the burden of ESKD.

## Notes

Supplement. **eMethods.** Detailed Methods **eTable.** Decomposition of the Total Association Between Body Mass Index and the Risk of ESKD into Direct and Indirect Associations Mediated by Fasting Glucose and by Fasting Triglycerides: Separate Mediation Models for Both Glucose and Triglycerides

## References

[1] Brück K, Stel VS, Gambaro G, ; European CKD Burden Consortium. CKD prevalence varies across the European general population. J Am Soc Nephrol. 2016;27(7):2135-2147. doi:10.1681/ASN.2015050542 26701975 PMC4926978

[2] Coresh J, Selvin E, Stevens LA, . Prevalence of chronic kidney disease in the United States. JAMA. 2007;298(17):2038-2047. doi:10.1001/jama.298.17.2038 17986697

[3] Hill NR, Fatoba ST, Oke JL, . Global prevalence of chronic kidney disease—a systematic review and meta-analysis. PLoS One. 2016;11(7):e0158765. doi:10.1371/journal.pone.0158765 27383068 PMC4934905

[4] Kovesdy CP, Furth SL, Zoccali C; World Kidney Day Steering Committee. Obesity and kidney disease: hidden consequences of the epidemic. Am J Nephrol. 2017;45(3):283-291. doi:10.1159/000458467 28178697

[5] Collaboration NCDRF; NCD Risk Factor Collaboration (NCD-RisC). Trends in adult body-mass index in 200 countries from 1975 to 2014: a pooled analysis of 1698 population-based measurement studies with 19·2 million participants. Lancet. 2016;387(10026):1377-1396. doi:10.1016/S0140-6736(16)30054-X 27115820 PMC7615134

[6] Garofalo C, Borrelli S, Minutolo R, Chiodini P, De Nicola L, Conte G. A systematic review and meta-analysis suggests obesity predicts onset of chronic kidney disease in the general population. Kidney Int. 2017;91(5):1224-1235. doi:10.1016/j.kint.2016.12.013 28187985

[7] Hsu CY, McCulloch CE, Iribarren C, Darbinian J, Go AS. Body mass index and risk for end-stage renal disease. Ann Intern Med. 2006;144(1):21-28. doi:10.7326/0003-4819-144-1-200601030-00006 16389251

[8] Zitt E, Pscheidt C, Concin H, Kramar R, Lhotta K, Nagel G. Anthropometric and metabolic risk factors for ESRD are disease-specific: results from a large population-based cohort study in Austria. PLoS One. 2016;11(8):e0161376. doi:10.1371/journal.pone.0161376 27537361 PMC4990261

[9] Zitt E, Pscheidt C, Concin H, . Long-term risk for end-stage kidney disease and death in a large population-based cohort. Sci Rep. 2018;8(1):7729. doi:10.1038/s41598-018-26087-z 29769597 PMC5955909

[10] Global Burden of Disease Study 2013 Collaborators. Global, regional, and national incidence, prevalence, and years lived with disability for 301 acute and chronic diseases and injuries in 188 countries, 1990-2013: a systematic analysis for the Global Burden of Disease Study 2013. Lancet. 2015;386(9995):743-800. doi:10.1016/S0140-6736(15)60692-4 26063472 PMC4561509

[11] Mortality GBD; GBD 2013 Mortality and Causes of Death Collaborators. Global, regional, and national age-sex specific all-cause and cause-specific mortality for 240 causes of death, 1990-2013: a systematic analysis for the Global Burden of Disease Study 2013. Lancet. 2015;385(9963):117-171. doi:10.1016/S0140-6736(14)61682-2 25530442 PMC4340604

[12] Mount PF, Juncos LA. Obesity-related CKD: when kidneys get the munchies. J Am Soc Nephrol. 2017;28(12):3429-3432. doi:10.1681/ASN.2017080850 29054854 PMC5698082

[13] Whaley-Connell A, Sowers JR. Obesity and kidney disease: from population to basic science and the search for new therapeutic targets. Kidney Int. 2017;92(2):313-323. doi:10.1016/j.kint.2016.12.034 28341271

[14] Artunc F, Schleicher E, Weigert C, Fritsche A, Stefan N, Häring HU. The impact of insulin resistance on the kidney and vasculature. Nat Rev Nephrol. 2016;12(12):721-737. doi:10.1038/nrneph.2016.145 27748389

[15] Câmara NO, Iseki K, Kramer H, Liu ZH, Sharma K. Kidney disease and obesity: epidemiology, mechanisms and treatment. Nat Rev Nephrol. 2017;13(3):181-190. doi:10.1038/nrneph.2016.191 28090083

[16] Whaley-Connell A, Sowers JR. Insulin resistance in kidney disease: is there a distinct role separate from that of diabetes or obesity? Cardiorenal Med. 2017;8(1):41-49. doi:10.1159/000479801 29344025 PMC5757598

[17] Simental-Mendía LE, Rodríguez-Morán M, Guerrero-Romero F. The product of fasting glucose and triglycerides as surrogate for identifying insulin resistance in apparently healthy subjects. Metab Syndr Relat Disord. 2008;6(4):299-304. doi:10.1089/met.2008.0034 19067533

[18] Guerrero-Romero F, Simental-Mendía LE, González-Ortiz M, . The product of triglycerides and glucose, a simple measure of insulin sensitivity: comparison with the euglycemic-hyperinsulinemic clamp. J Clin Endocrinol Metab. 2010;95(7):3347-3351. doi:10.1210/jc.2010-0288 20484475

[19] Ulmer H, Kelleher C, Diem G, Concin H. Long-term tracking of cardiovascular risk factors among men and women in a large population-based health system: the Vorarlberg Health Monitoring & Promotion Programme. Eur Heart J. 2003;24(11):1004-1013. doi:10.1016/S0195-668X(03)00170-2 12788300

[20] Zitt E, Fischer A, Lhotta K, Concin H, Nagel G. Sex- and age-specific variations, temporal trends and metabolic determinants of serum uric acid concentrations in a large population-based Austrian cohort. Sci Rep. 2020;10(1):7578. doi:10.1038/s41598-020-64587-z 32371883 PMC7200724

[21] Wimmer F, Oberaigner W, Kramar R, Mayer G. Regional variability in the incidence of end-stage renal disease: an epidemiological approach. Nephrol Dial Transplant. 2003;18(8):1562-1567. doi:10.1093/ndt/gfg184 12897095

[22] World Medical Association. World Medical Association Declaration of Helsinki: ethical principles for medical research involving human subjects. JAMA. 2013;310(20):2191-2194. doi:10.1001/jama.2013.281053 24141714

[23] von Elm E, Altman DG, Egger M, Pocock SJ, Gøtzsche PC, Vandenbroucke JP; STROBE Initiative. The Strengthening the Reporting of Observational Studies in Epidemiology (STROBE) statement: guidelines for reporting observational studies. Prev Med. 2007;45(4):247-251. doi:10.1016/j.ypmed.2007.08.012 17950122

[24] World Health Organization. BMI classification. Accessed February 21, 2020. https://apps.who.int/bmi/index.jsp?introPage=intro_3.html

[25] VanderWeele TJ. Causal mediation analysis with survival data. Epidemiology. 2011;22(4):582-585. doi:10.1097/EDE.0b013e31821db37e 21642779 PMC3109321

[26] Greenland S, Pearl J, Robins JM. Causal diagrams for epidemiologic research. Epidemiology. 1999;10(1):37-48. doi:10.1097/00001648-199901000-00008 9888278

[27] Lange T, Hansen KW, Sørensen R, Galatius S. Applied mediation analyses: a review and tutorial. Epidemiol Health. 2017;39:e2017035. doi:10.4178/epih.e2017035 29121709 PMC5723912

[28] VanderWeele TJ. Mediation analysis: a practitioner’s guide. Annu Rev Public Health. 2016;37:17-32. doi:10.1146/annurev-publhealth-032315-021402 26653405

[29] Valeri L, Vanderweele TJ. Mediation analysis allowing for exposure-mediator interactions and causal interpretation: theoretical assumptions and implementation with SAS and SPSS macros. Psychol Methods. 2013;18(2):137-150. doi:10.1037/a0031034 23379553 PMC3659198

[30] Valeri L, VanderWeele TJ. SAS macro for causal mediation analysis with survival data. Epidemiology. 2015;26(2):e23-e24. doi:10.1097/EDE.0000000000000253 25643116

[31] Foster MC, Hwang SJ, Larson MG, . Overweight, obesity, and the development of stage 3 CKD: the Framingham Heart Study. Am J Kidney Dis. 2008;52(1):39-48. doi:10.1053/j.ajkd.2008.03.003 18440684 PMC2531220

[32] Gelber RP, Kurth T, Kausz AT, . Association between body mass index and CKD in apparently healthy men. Am J Kidney Dis. 2005;46(5):871-880. doi:10.1053/j.ajkd.2005.08.015 16253727

[33] Kramer H, Luke A, Bidani A, Cao G, Cooper R, McGee D. Obesity and prevalent and incident CKD: the Hypertension Detection and Follow-Up Program. Am J Kidney Dis. 2005;46(4):587-594. doi:10.1053/j.ajkd.2005.06.007 16183412

[34] Xu H, Kuja-Halkola R, Chen X, Magnusson PKE, Svensson P, Carrero JJ. Higher body mass index is associated with incident diabetes and chronic kidney disease independent of genetic confounding. Kidney Int. 2019;95(5):1225-1233. doi:10.1016/j.kint.2018.12.019 30898340

[35] Lu JL, Molnar MZ, Naseer A, Mikkelsen MK, Kalantar-Zadeh K, Kovesdy CP. Association of age and BMI with kidney function and mortality: a cohort study. Lancet Diabetes Endocrinol. 2015;3(9):704-714. doi:10.1016/S2213-8587(15)00128-X 26235959 PMC4547884

[36] Iseki K, Ikemiya Y, Kinjo K, Inoue T, Iseki C, Takishita S. Body mass index and the risk of development of end-stage renal disease in a screened cohort. Kidney Int. 2004;65(5):1870-1876. doi:10.1111/j.1523-1755.2004.00582.x 15086929

[37] Munkhaugen J, Lydersen S, Widerøe TE, Hallan S. Prehypertension, obesity, and risk of kidney disease: 20-year follow-up of the HUNT I study in Norway. Am J Kidney Dis. 2009;54(4):638-646. doi:10.1053/j.ajkd.2009.03.023 19515474

[38] Vivante A, Golan E, Tzur D, . Body mass index in 1.2 million adolescents and risk for end-stage renal disease. Arch Intern Med. 2012;172(21):1644-1650. doi:10.1001/2013.jamainternmed.85 23108588 PMC4941233

[39] Reaven GM. Banting lecture 1988: role of insulin resistance in human disease. Diabetes. 1988;37(12):1595-1607. doi:10.2337/diab.37.12.1595 3056758

[40] Chen J, Muntner P, Hamm LL, . The metabolic syndrome and chronic kidney disease in US adults. Ann Intern Med. 2004;140(3):167-174. doi:10.7326/0003-4819-140-3-200402030-00007 14757614

[41] Mykkänen L, Zaccaro DJ, Wagenknecht LE, Robbins DC, Gabriel M, Haffner SM. Microalbuminuria is associated with insulin resistance in nondiabetic subjects: the insulin resistance atherosclerosis study. Diabetes. 1998;47(5):793-800. doi:10.2337/diabetes.47.5.793 9588452

[42] Jha V, Garcia-Garcia G, Iseki K, . Chronic kidney disease: global dimension and perspectives. Lancet. 2013;382(9888):260-272. doi:10.1016/S0140-6736(13)60687-X 23727169

[43] Radhakrishnan J, Remuzzi G, Saran R, ; CDC-CKD Surveillance Team; European CKD Burden Consortium; CKD.QLD group. Taming the chronic kidney disease epidemic: a global view of surveillance efforts. Kidney Int. 2014;86(2):246-250. doi:10.1038/ki.2014.190 24897034 PMC4593485

[44] Coen PM, Tanner CJ, Helbling NL, . Clinical trial demonstrates exercise following bariatric surgery improves insulin sensitivity. J Clin Invest. 2015;125(1):248-257. doi:10.1172/JCI78016 25437877 PMC4382227

[45] Reed MA, Pories WJ, Chapman W, . Roux-en-Y gastric bypass corrects hyperinsulinemia implications for the remission of type 2 diabetes. J Clin Endocrinol Metab. 2011;96(8):2525-2531. doi:10.1210/jc.2011-0165 21593117

[46] Shulman A, Peltonen M, Sjöström CD, . Incidence of end-stage renal disease following bariatric surgery in the Swedish Obese Subjects Study. Int J Obes (Lond). 2018;42(5):964-973. doi:10.1038/s41366-018-0045-x 29568103 PMC6019553

[47] Kramer H, Gutiérrez OM, Judd SE, . Waist circumference, body mass index, and ESRD in the REGARDS (Reasons for Geographic and Racial Differences in Stroke) study. Am J Kidney Dis. 2016;67(1):62-69. doi:10.1053/j.ajkd.2015.05.023 26187471 PMC5628031

[48] Elsayed EF, Sarnak MJ, Tighiouart H, . Waist-to-hip ratio, body mass index, and subsequent kidney disease and death. Am J Kidney Dis. 2008;52(1):29-38. doi:10.1053/j.ajkd.2008.02.363 18511168 PMC4052757

[49] Madero M, Katz R, Murphy R, . Comparison between different measures of body fat with kidney function decline and incident CKD. Clin J Am Soc Nephrol. 2017;12(6):893-903. doi:10.2215/CJN.07010716 28522656 PMC5460706

