# Supplementary material for: The Triglyceride-Glucose Index and Obesity-Related Risk of End-Stage Kidney Disease in Austrian Adults
Source: JAMA Netw Open. 2021 Mar 31;4(3):e212612. doi: 10.1001/jamanetworkopen.2021.2612 (PMC8013829; doi:10.1001/jamanetworkopen.2021.2612)
Supplement: Supplement. — eMethods. Detailed Methods eTable. Decomposition of the Total Association Between Body Mass Index and the Risk of ESKD into Direct and Indirect Associations Mediated by Fasting Glucose and by Fasting Triglycerides: Separate Mediation Models for Both Glucose and Triglycerides [file jamanetwopen-e212612-s001.pdf]

Supplementary Online Content

Fritz J, Brozek W, Concin H, et al. The triglyceride-glucose index and obesity-related risk of end-stage kidney disease in Austrian adults. *JAMA Netw Open*. 2021;4(3):e212612. doi:10.1001/jamanetworkopen.2021.2612

eMethods. Detailed Methods

eTable. Decomposition of the Total Association Between Body Mass Index and the Risk of ESKD into Direct and Indirect Associations Mediated by Fasting Glucose and by Fasting Triglycerides: Separate Mediation Models for Both Glucose and Triglycerides

This supplementary material has been provided by the authors to give readers additional information about their work.

© 2021 Fritz J et al. *JAMA Network Open.*

eMethods. Detailed Methods

We did not adjust for blood pressure, cholesterol, or uric acid because, under the plausible assumption that these variables do not causally affect BMI, they do not qualify as confounders of the association between BMI and the risk of ESKD. Rather, they act as other potential mediators in the association between BMI and ESKD according to our causal model (**Figure 1**), and should therefore not be adjusted for.[1]

The presence of independent associations both between exposure and mediator, and between mediator and outcome has been described as a necessary condition for the mediator to convey the exposure effect to the outcome.[2] Therefore, the observed associations between BMI and the TyG index, as well as (BMI-adjusted) between the TyG index and the risk of ESKD (represented by the two arrows BMI→TyG index, and TyG index→ESKD in the directed acyclic graph (DAG) of **Figure 1**) indicate the presence of mediation of the association between BMI and ESKD risk through the TyG index.

1. Greenland S, Pearl J, Robins JM. Causal diagrams for epidemiologic research. *Epidemiology.* 1999;10(1):37-48.
2. Baron RM, Kenny DA. The moderator-mediator variable distinction in social psychological research: conceptual, strategic, and statistical considerations. *J Pers Soc Psychol.* 1986;51(6):1173-1182.

© 2021 Fritz J et al. *JAMA Network Open.*

eTable: Decomposition of the total association between body mass index and the risk of ESKD into direct and indirect associations mediated by fasting glucose and by fasting triglycerides: separate mediation models for both glucose and triglycerides.

Group	ESKD incident cases/persons (N)	Total association HR (95% CI) p-value	Indirect association HR (95% CI) p-value	Direct association HR (95% CI) p-value	Proportion mediated (95% CI)
**Mediation through fasting glucose**					
**Total population**	454/176 420	1.51 (1.38-1.66)<br><0.001	1.05 (1.04-1.06)<br><0.001	1.44 (1.31-1.58)<br><0.001	11.6% (8.6%-14.6%)
**Participants with baseline fasting glucose <126 mg/dL only**	365/169 909	1.46 (1.31-1.63)<br><0.001	1.00 (0.98-1.02)<br>0.91	1.46 (1.31-1.63)<br><0.001	-0.3% (-4.9%-4.4%)
**Mediation through fasting triglycerides**					
**Total population**	454/176 420	1.57 (1.43-1.72)<br><0.001	1.05 (1.04-1.06)<br><0.001	1.49 (1.36-1.64)<br><0.001	10.8% (7.8%-13.8%)
**Participants with baseline fasting glucose <126 mg/dL only**	365/169 909	1.45 (1.30-1.63)<br><0.001	1.04 (1.03-1.06)<br><0.001	1.39 (1.24-1.56)<br><0.001	11.1% (6.6%-15.6%)

Decomposition of total associations into natural indirect and natural direct associations was done according to the 2-stage regression method proposed by VanderWeele, and performed with the SAS macro provided by Valeri and VanderWeele. Confidence intervals were calculated according to the delta method procedure. All models were adjusted for age, sex, and smoking status as depicted in the DAG in Figure 1.

HRs given per 5 kg/m² increase.

Abbreviations: CI, confidence interval; DAG, directed acyclic graph; HR, hazard ratio

© 2021 Fritz J et al. *JAMA Network Open.*
